# Broad-spectrum antiviral agents: secreted phospholipase A_2_ targets viral envelope lipid bilayers derived from the endoplasmic reticulum membrane

**DOI:** 10.1038/s41598-017-16130-w

**Published:** 2017-11-21

**Authors:** Ming Chen, Chie Aoki-Utsubo, Masanori Kameoka, Lin Deng, Yutaka Terada, Wataru Kamitani, Kei Sato, Yoshio Koyanagi, Makoto Hijikata, Keiko Shindo, Takeshi Noda, Michinori Kohara, Hak Hotta

**Affiliations:** 1 Department of Vaccine and Drug Development, Graduate School of Health Sciences, Kobe University, Kobe, 650–0047 Japan; 2 Department of International Health, Graduate School of Health Sciences, Kobe University, Kobe, 654–0147 Japan; 3 Division of Infectious Disease Control, Graduate School of Medicine, Kobe University, Kobe, 650–0017 Japan; 4 Laboratory of Clinical Research on Infectious Diseases, Research Institute for Microbial Diseases, Osaka University, Suita, Osaka, 565–0871 Japan; 5 Laboratory of Systems Virology, Institute for Frontier Life and Medical Sciences, Kyoto University, Kyoto, 606–8507 Japan; 6 CREST, Japan Science and Technology Agency, Saitama, 322-0012 Japan; 7 Laboratory of Tumour Viruses, Institute for Frontier Life and Medical Sciences, Kyoto University, Kyoto, 606-8507 Japan; 8 Laboratory of Ultrastructural Virology, Institute for Frontier Life and Medical Sciences, Kyoto University, Kyoto, 606-8507 Japan; 9 Infectious Disease Regulation Project, Tokyo Metropolitan Institute of Medical Science, Tokyo, 156-8506 Japan

## Abstract

Hepatitis C virus (HCV), dengue virus (DENV) and Japanese encephalitis virus (JEV) belong to the family *Flaviviridae*. Their viral particles have the envelope composed of viral proteins and a lipid bilayer acquired from budding through the endoplasmic reticulum (ER). The phospholipid content of the ER membrane differs from that of the plasma membrane (PM). The phospholipase A_2_ (PLA_2_) superfamily consists of a large number of members that specifically catalyse the hydrolysis of phospholipids at a particular position. Here we show that the CM-II isoform of secreted PLA_2_ obtained from *Naja mossambica mossambica* snake venom (CM-II-sPLA_2_) possesses potent virucidal (neutralising) activity against HCV, DENV and JEV, with 50% inhibitory concentrations (IC_50_) of 0.036, 0.31 and 1.34 ng/ml, respectively. In contrast, the IC_50_ values of CM-II-sPLA_2_ against viruses that bud through the PM (Sindbis virus, influenza virus and Sendai virus) or *trans*-Golgi network (TGN) (herpes simplex virus) were >10,000 ng/ml. Moreover, the 50% cytotoxic (CC_50_) and haemolytic (HC_50_) concentrations of CM-II-sPLA_2_ were >10,000 ng/ml, implying that CM-II-sPLA_2_ did not significantly damage the PM. These results suggest that CM-II-sPLA_2_ and its derivatives are good candidates for the development of broad-spectrum antiviral drugs that target viral envelope lipid bilayers derived from the ER membrane.

## Introduction

Cellular membrane compartments can be categorised into two groups; the first group consists of the endoplasmic reticulum (ER), nuclear envelope, lipid droplets and *cis*-Golgi (ER-NE-*cis*-Golgi lipid territory) and the second group consists of the *trans*-Golgi, plasma membrane (PM) and endosomes (*trans*-Golgi-PM-EE membrane territory)^1–3^. The ER-NE-*cis*-Golgi membranes have lipid packing defects, whereas the *trans*-Golgi-PM-EE membranes show tight packing of phospholipids^3^. Additionally, the phospholipid contents of the ER-NE-*cis*-Golgi membranes differ from those of the *trans*-Golgi-PM-EE membranes. Enveloped viruses acquire their envelope lipid bilayers from host cellular membranes^4^. Consequently, the phospholipid contents of viruses budding through the ER-NE-*cis*-Golgi membranes differ from those of viruses budding through the *trans*-Golgi-PM-EE membranes^5–7^. Moreover, the physicochemical characteristics, such as thickness and sturdiness, differ between the ER-NE-*cis*-Golgi and the *trans*-Golgi-PM-EE membranes^8,9^. Moreover, the sensitivity to hydrolysis by the secreted phospholipase A_2_ (sPLA_2_) enzyme has been reported to differ depending on the lipid composition and overall structure^10^.

Hepatitis C virus (HCV), dengue virus (DENV), Japanese encephalitis virus (JEV) and yellow fever virus (YFV), which belong to the family *Flaviviridae*, bud through the ER membrane to acquire their envelopes^11–13^. Conversely, influenza A virus (FLUAV) buds through the PM^14^, and its phospholipid content differs from that of DENV^5,6^. sPLA_2_ obtained from a venomous snake was reported to possess potent virucidal (neutralising) activity against DENV and YFV by disrupting the viral envelope lipid bilayers^15,16^. Human sPLA_2_ also shows virucidal activity against human immunodeficiency virus (HIV)^17^, which is known to bud through the PM^18^. Moreover, sPLA_2_s obtained from the venom of bees and another snake (*Naja mossambica mossambica*) were reported to inhibit the entry of HIV into host cells without disrupting the viral envelope^19^. While examining plant and animal substances for their neutralising activity against HCV^20,21^, we became interested in testing a number of sPLA_2_s from different sources regarding the specificity against different viruses.

Members of the large sPLA_2_ family show highly diverse sequence variation and functional characteristics and can be divided into 10 groups and 18 subgroups^22^. sPLA_2_ obtained from *N*. *m*. *mossambica* venom belongs to subgroup IA and is further subdivided into at least 6 isoforms, including CM-I, -II and –III^23–26^. Subgroup IB includes the pancreatic sPLA_2_s of humans, bovines and porcines. Groups III and XIV include honeybee venom sPLA_2_ and bacterial sPLA_2_, respectively. In the present study, we examined the possible virucidal activity of those sPLA_2_s against a panel of viruses. We found that the sPLA_2_ CM-II isoform from *N*. *m*. *mossambica* (CM-II-sPLA_2_) showed potent virucidal activity against HCV, DENV and JEV.

## Results

### CM-II-sPLA_2_ possesses potent virucidal activity against HCV, DENV and JEV, which bud through the ER membrane, but not against other viruses that bud through the PM

First, we tested the possible direct virucidal activity of CM-II-sPLA_2_ (UniProtKB-P00603 [PA2B2_NAJMO]) against infectious particles of a panel of viruses. Each virus was incubated with various concentrations of CM-II-sPLA_2_ or medium as a control at 37 °C for 1 h and then inoculated onto Huh7it-1 cells. After 1 h of virus adsorption, the residual virus was removed by washing the cells with medium and the cells were cultured for 24 h. The cells were subjected to fluorescent antibody (FA) staining with respective antiviral antibodies to determine the number of virus-infected cells, which represented the inoculum’s viral titres (cell-infecting units [CIU]/ml). In some experiments, a plaque assay and 50% tissue culture infectious dose (TCID_50_) assay were performed to determine the viral titres of the inocula (plaque-forming units [PFU]/ml and TCID_50_/ml, respectively). The 50% inhibitory concentration (IC_50_) was calculated based on the percent reduction of the initial viral titre.

The results demonstrated that CM-II-sPLA_2_ efficiently neutralised the infectivity of HCV, DENV and JEV, with 50%-inhibitory concentrations (IC_50_) of 0.036 ng/ml (0.003 nM), 0.31 ng/ml (0.023 nM) and 1.34 ng/ml (0.10 nM), respectively (Table 1). The dose-dependent inhibition of the viruses by CM-II-sPLA_2_ is shown in Supplementary Fig. S1. HCV, DENV and JEV belong to the family *Flaviviridae* and are known to bud through the ER membrane^11–13^. Conversely, CM-II-sPLA_2_ even at a dose of 10,000 ng/ml did not exert significant virucidal activity against Sindbis virus (SINV; *Togaviridae*)^27^, influenza A virus (FLUAV; *Orthomyxoviridae*) and Sendai virus (SeV; *Paramyxoviridae*)^28^, which are known to bud through the PM, or herpes simplex virus type 1 (HSV-1; *Herpesviridae*), which buds through the *trans*-Golgi network (TGN)^29^. Vesicular stomatitis New Jersey virus (VSNJV; *Rhabdoviridae*), which also buds through the PM^30^, showed weak sensitivity to CM-II-sPLA_2_, with an IC_50_ value of 2,300 ng/ml. The weak CM-II-sPLA_2_ sensitivity of VSNJV compared to the insensitivity of SINV, FLUAV and HSV-1 could be attributable to the bullet-shape configuration of VSNJV, which might harbour lipid packing defects at the bottom edge of the bullet-shape particles. CM-II-sPLA_2_ inhibited HIV infection with an IC_50_ of 5.4 ng/ml. This result is consistent with a previous observation that sPLA_2_ obtained from bee and snake venoms inhibited HIV entry into host cells without disrupting the HIV particles^19^.

**Table 1:** Virucidal activity of CM-II-sPLA_2_ against different viruses.

Virus	Family	Site of virus budding	IC_50_ (ng/ml)
HCV	*Flaviviridae*	ER	0.036 ± 0.004
DENV	*Flaviviridae*	ER	0.31 ± 0.07
JEV	*Flaviviridae*	ER	1.34 ± 0.21
MERS-CoV	*Coronaviridae*	ERGIC	10,000
SINV	*Togaviridae*	PM	>10,000
FLUAV	*Orthomyxoviridae*	PM	>10,000
SeV	*Paramyxoviridae*	PM	>10,000
VSNJV	*Rhabdoviridae*	PM	2,300 ± 1,333
HIV-1	*Retroviridae*	PM	5.4
HSV-1	*Herpesviridae*	TGN	>10,000
EMCV	*Picornaviridae*	(Non-enveloped)	>10,000
CV-B3	*Picornaviridae*	(Non-enveloped)	>10,000

Data are presented as the average ± SEM (n = 3 to 5).

We also tested Middle East respiratory syndrome coronavirus (MERS-CoV), which is a member of family *Coronaviridae* that is known to bud through the ER-Golgi intermediate compartment (ERGIC)^31^. The ERGIC constitutes part of the ER-NE-*cis*-Golgi territory and therefore is considered to have similar characteristics with the ER. Rather unexpectedly, we found that CM-II-sPLA_2_ exerted only marginal, if any, virucidal activity against MERS-CoV, with an IC_50_ of 10,000 ng/ml. We assume that the exceptionally large petal-shaped spikes that project approximately 20 nm from the virion envelope, which is a characteristic feature of members of *Coronaviridae* ^31^, interfere with the access of CM-II-sPLA_2_ to the envelope lipid bilayer. Alternatively, a dose-dependent inhibition pattern (see panel (d) in Supplementary Fig. S1) may imply the possibility that there are two groups of MERS-CoV particles: one that is sensitive to 100 ng/ml of CM-II-sPLA_2_ and another that is resistant to 10,000 ng/ml of CM-II-sPLA_2_. The latter group may represent viral particles that have budded through the PM, as reported previously^32^. Further studies are needed to elucidate the issue.

As expected, CM-II-sPLA_2_ did not neutralize the infectivity of encephalomyocarditis virus (EMCV) and coxsackievirus B3 (CV-B3), which belong to family *Picornaviridae* and do not possess envelopes^33^, even at the 10,000 ng/ml dose.

### CM-II-sPLA_2_ does not inhibit viral replication of HCV and DENV when added to the cells after viral entry

**Figure 1:**
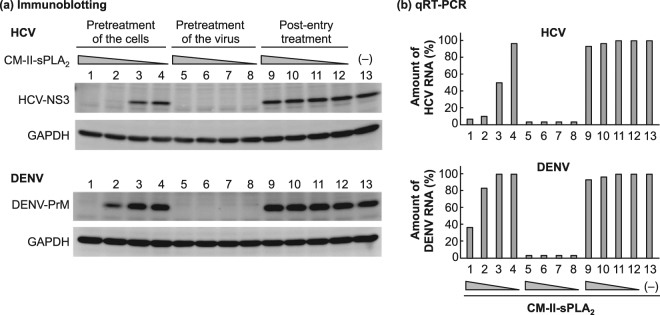
Time-of-addition experiments using CM-II-sPLA_2_ against HCV and DENV. (**a**) Immunoblotting analysis. The HCV NS3 (upper panel) and DENV PrM (lower panel) expression levels were examined by immunoblotting. (i) Pretreatment of the cells: Huh7it-1 cells were treated with decreasing concentrations of CM-II-sPLA_2_ (1,000, 100, 10 and 1 ng/ml) for 1 h. Then, the cells were inoculated with HCV or DENV in the absence of CM-II-sPLA_2_ for another 1 h and cultured for 24 h in the absence of CM-II-sPLA_2_. (ii) Pretreatment of the virus: HCV and DENV were incubated with CM-II-sPLA_2_ for 1 h, and the mixtures were inoculated onto Huh7it-1 cells. After 1 h, the cells were cultured for 24 h in the absence of CM-II-sPLA_2_. (iii) Post-entry treatment: Huh7it-1 cells were inoculated with HCV or DENV in the absence of CM-II-sPLA_2_. After 1 h, the cells were cultured for 24 h in the presence of CM-II-sPLA_2_. (–), Untreated control. The full-length gels and blots are shown in Supplementary Figs S2 and S3. (**b**) qRT-PCR analysis. The HCV RNA (upper panel) and DENV RNA (lower panel) levels in cells prepared under the same conditions described in (**a**) were quantified by qRT-PCR. (–), Untreated control. Data are presented as the percentage of the untreated control.

Time-of-addition experiments were performed to examine whether CM-II-sPLA_2_ inhibited viral replication after the virus entered the host cells. The results showed that post-inoculation (post-entry) treatment of HCV- and DENV-infected cells with CM-II-sPLA_2_ (1 to 1,000 ng/ml) for 24 h did not reduce the number of infected cells (see Supplementary Table S1). We examined viral protein synthesis and RNA replication in the infected cells under the same experimental conditions. The results demonstrated that whereas pretreatment of the virus with CM-II-sPLA_2_ at a dose as low as 1.0 ng/ml almost completely inhibited HCV and DENV infection, post-entry treatment of virus-infected cells with CM-II-sPLA_2_ even at the 1,000 ng/ml dose did not inhibit viral protein synthesis (Fig. 1a) or RNA replication (Fig. 1b). Notably, pretreatment of cells with CM-II-sPLA_2_ at 100 and 1,000 ng/ml doses prior to viral inoculation resulted in substantial inhibition of viral replication. This result is consistent with the result shown in Supplementary Table S1. Determining whether CM-II-sPLA_2_ that bound to (or was taken up by) the cells was released into the medium during viral inoculation to neutralise the infectivity of the inoculum or whether CM-II-sPLA_2_ treatment induced downregulation of the viral receptor(s) and/or upregulation of the antiviral status in the cells requires further investigation. In any case, these results confirmed the potent virucidal activity of CM-II-sPLA_2_ against HCV and DENV.

### CM-II-sPLA_2_ does not mediate *in vitro* cytotoxicity or haemolytic activity

We also tested the cytotoxicity and haemolytic activity of CM-II-sPLA_2_. Uninfected Huh7it-1 cells were incubated with various concentrations of CM-II-sPLA_2_ at 37 °C for 24 h. Cell viability was determined using a WST-1 assay and lactate dehydrogenase (LDH) release test, and the 50% cytotoxic concentrations (CC_50_) were calculated. The haemolysis assay was performed by incubating human red blood cells (RBCs) with CM-II-sPLA_2_ at 37 °C for 1 h, and the 50% haemolytic concentration (HC_50_) was calculated. CM-II-sPLA_2_ even at the 10,000 ng/ml dose mediated only marginal, if any, cytotoxicity or haemolytic activity (Table 2). The selectivity indices (CC_50_/IC_50_) of CM-II-sPLA_2_ against HCV and DENV were >270,000 and >32,000, respectively.

**Table 2:** Virucidal activity of various sPLA_2_s from different sources.

sPLA_2_	IC_50_ (ng/ml)								CC_50_ or HC_50_ (ng/ml)		
	HCV	DENV	JEV	SINV	FLUAV	SeV	VSNJV	HSV-1	WST-1	LDH	Haemolysis
CM-II-sPLA_2_	0.036^a)^	0.31^a)^	1.34^a)^	>10,000^a)^	>10,000^a)^	>10,000^a)^	2,300^a)^	>10,000^a)^	>10,000	>10,000	>10,000
*A*. *mellifera*	117 ± 43	183 ± 38	49 ± 13	>10,000	>10,000	>10,000	>10,000	>10,000	>10,000	>10,000	>10,000
*S*. *violaceoruber*	>10,000	>10,000	>10,000	>10,000	>10,000	>10,000	>10,000	>10,000	>10,000	>10,000	>10,000
Bovine pancreas	>10,000	>10,000	>10,000	>10,000	>10,000	>10,000	>10,000	>10,000	>10,000	>10,000	>10,000
Porcine pancreas	>10,000	>10,000	>10,000	>10,000	>10,000	>10,000	>10,000	>10,000	>10,000	>10,000	>10,000

Data are presented as the average ± SEM (n = 3 to 5).

(a) The same IC_50_ values as shown in Table 1.

### sPLA_2_ from bee venom (group III) possesses moderate virucidal activity against HCV, DENV and JEV, whereas sPLA_2_ from bovine and porcine pancreas (subgroup IB) and bacteria (group XIV) do not exert significant virucidal activity

Next, we tested the possible virucidal activity of sPLA_2_s from other groups and subgroups. The results showed that sPLA_2_s obtained from bovine and porcine pancreas (subgroup IB) or from *Streptomyces violaceoruber* (group XIV) did not neutralise HCV, DENV or JEV even at the 10,000 ng/ml dose (Table 2). However, sPLA_2_ obtained from the venom of the honeybee *Apis mellifera* (group III) showed a moderate degree of virucidal activity against HCV, DENV and JEV, with IC_50_ values of 117, 183 and 49 ng/ml, respectively, while mediating no detectable cytotoxicity or haemolytic activity even at the 10,000 ng/ml dose (Table 2).

### The sPLA_2_ inhibitor manoalide counteracts the antiviral activity of CM-II-sPLA_2_ against HCV and DENV

Manoalide is known to irreversibly inhibit the enzymatic activity of sPLA_2_^22,34^. We tested the possible inhibitory effect of manoalide on the virucidal activity of CM-II-sPLA_2_. CM-II-sPLA_2_ (2 μg/ml) was incubated in the presence or absence of manoalide (10 μg/ml) in a buffer containing 10 mM HEPES (pH 7.2) and 1 mM CaCl_2_ at 41 °C for 1 h. Then, the manoalide-treated CM-II-sPLA_2_ or untreated control was tested for virucidal activity against HCV and DENV. The results clearly showed that the virucidal activity of CM-II-sPLA_2_ against HCV and DENV was markedly inhibited by manoalide (Table 3). This finding suggests that the virucidal activity of CM-II-sPLA_2_ against HCV and DENV is closely associated with its enzymatic activity.

**Table 3:** Inhibition of the virucidal activity of CM-II-sPLA_2_ by manoalide.

Virus	IC_50_ of CM-II-sPLA_2_ (ng/ml) treated with:		Fold difference
	Manoalide	Control	
HCV	6.0 ± 4.1	0.035 ± 0.005	171
DENV	18.4 ± 13.6	0.26 ± 0.02	71

Data are presented as the average ± SEM (n = 2).

## Discussion

The PLA_2_ superfamily is currently classified into six types, namely, sPLA_2_, Ca^2+^-dependent cytosolic cPLA_2_, Ca^2+^-independent cytosolic iPLA_2_, platelet-activating factor acetyl hydrolase PAF-AH, lysosomal LPLA_2_ and adipose-specific AdPLA_2_^22^. More than one-third of the members of the PLA_2_ superfamily belong to sPLA_2_, which is further divided into 10 groups (e.g., I, II, III, and XIV) and 18 subgroups (e.g., IA, IB, and IIA). In addition to their highly diverse sequence variations, sPLA_2_s show functional variations and are involved in a wide range of biological functions and disease occurrence through lipid metabolism and signalling^35^. Moreover, the sPLA_2_s enzymes catalyse the hydrolysis at the *sn*−2 position of the glycerol backbone of phospholipids^22^.

In the present study, we have demonstrated that CM-II-sPLA_2_ belonging to subgroup IA^22–26^ possesses potent virucidal (neutralising) activity against HCV, DENV and JEV particles, which bud through the ER, but does not neutralise the infectivity of SINV, FLUAV, SeV and HSV-1, which bud through the PM or TGN (Table 1). The potent virucidal activity against HCV and DENV was markedly inhibited by a specific sPLA_2_ inhibitor (Table 3). These results suggest that CM-II-sPLA_2_ selectively disrupts viral envelope lipid bilayers derived from the ER through phospholipid hydrolysis, although we could not obtain the direct evidence by negative staining electron microscopic analysis of the viral particles.

The phospholipid content of the ER-NE-*cis*-Golgi membranes differs from that of the *trans*-Golgi-PM-EE membranes^1–3^, which basically accounts for the difference in the phospholipid contents of the viral envelope between viruses budding through the ER-NE-*cis*-Golgi membranes and those budding through the *trans*-Golgi-PM-EE membranes^4–6^. For example, viruses budding through the ER (HCV and bovine viral diarrhea virus [BVDV]) were reported to contain more phosphatidylcholine and less phosphatidylserine than viruses budding through the PM (FLUAV, VSV, HIV and Semliki Forest virus [SFV])^7^. This difference is mainly a reflection of the intrinsic chemical differences between the ER and PM^1–3,8,9^. Additionally, the biophysical thickness and sturdiness differ between the ER and the PM, with the former being thin and less sturdy with lipid packing defects in the lipid bilayers and the latter being thick and sturdier with tight lipid packaging^3,8,9^. Moreover, the envelope proteins of viruses belonging to the family *Flaviviridae* lie flat across the surface of the lipid bilayer of the virion, which allows sPLA_2_ to access to lipid bilayers more easily than the lipid bilayers of other enveloped viruses with protruding envelope proteins. These physicochemical and structural differences in lipid bilayers and whole envelopes would account for the specificity of the virucidal activity of CM-II-sPLA_2_ against different viruses. In this sense, CM-II-sPLA_2_, and possibly other sPLA_2_s can be used to examine the budding site(s) of infectious particles of various viruses in more detail.

We also need to take into consideration that the phospholipid composition of the viral envelope varies even among different viral species in a given viral family, e.g., HCV, DENV, BVDV and West Nile virus (WNV) of the family *Flaviviridae* ^36^. Since the substrate specificity of each sPLA_2_ should differ depending on its intrinsic properties, the specificity of the virucidal activity of each sPLA_2_ against a given virus is most likely determined by a particular composition of phospholipids and their distributions on the viral envelope. Moreover, the lipid compositions of HCV, WNV and BVDV, which bud through the ER membrane, were reported to differ from that of a typical ER membrane^7,36–38^. Thus, viruses probably modify their intracellular microenvironments, including the physicochemical conditions of the host cell membranes to facilitate progeny virus production.

CM-II-sPLA_2_ has been shown to mediate neurotoxicity *in vivo*, although to a lesser extent than the CM-III isoform of *N*. *m*. *mossambica* sPLA_2_^23,25^. The CM-III isoform and other neurotoxic sPLA_2_s possess not only PM-disrupting activity but a high binding affinity for the N-type (neuronal-type) receptor, the latter of which is closely associated with neurotoxicity^39^. Therefore, CM-II-sPLA_2_ can conceivably be taken up by neural cells through binding to a receptor (e.g., the N-type receptor) and disrupt certain intracellular membrane compartment(s) inside the neuronal cells to mediate neurotoxicity^40,41^.

Taking advantage of the intrinsic differences in the physicochemical properties of the membrane lipid bilayers between viruses and host cells as well as the specific interactions between sPLA_2_s and their receptors, a broad-spectrum antiviral sPLA_2_(s) can be developed to target viral envelope lipid bilayers derived from the ER without targeting the lipid bilayers of host cell membranes. A CM-II-sPLA_2_ mutant(s) that has a weaker or no binding affinity for the N-type receptor but retains potent virucidal activity would be a good candidate for development of a novel antiviral drug.

## Methods

### Chemicals

CM-II-sPLA_2_ (P7778) and other sPLA_2_s obtained from *A*. *mellifera* honeybee venom (P9279), bovine pancreas (P8913) and *S*. *violaceoruber* (P8685) were purchased from Sigma-Aldrich. sPLA_2_ from porcine pancreas was a kind gift from Sanyo Fine (Osaka, Japan). Manoalide was purchased from Abcam.

### Viruses and cells

HCV (J6/JFH-1 strain)^20,21,42,43^, DENV (Trinidad 1751 strain)^44,45^, JEV (Nakayama strain)^46^, SINV^47^, FLUAV (A/Udorn/307/72 [H3N2])^21^, SeV (Fushimi strain)^48^, VSNJV^46^, HSV-1 (CHR3 strain)^49^, EMCV (DK-27 strain)^46^, CV-B3 (Nancy strain)^50^, HIV-1 (NLCSFV3 strain)^51^ and MERS-CoV (EMC2012 strain)^52^ were described previously. HCV, DENV, JEV, SINV, VSNJV, HSV-1, EMCV and CV-B3 were prepared in Huh7it-1 cells^53^. FLUAV, HIV-1 and MERS-CoV were prepared in MDCK, HEK293T and Vero cells, respectively. SeV was prepared in embryonated eggs. The infectivity of all viruses except HIV-1 and MERS-CoV was determined on Huh7it-1 cells. Plaque assays were performed for SINV, VSNJV and EMCV by cultivating the virus-inoculated cells for 2 to 4 days under overlay medium containing 1% methylcellulose as described previously^46^ and the viral titres were expressed as PFU/ml. For the remaining viruses except HIV-1, infectivity was determined by a FA method using specific antibodies against the respective viruses, and viral titres were expressed as CIU/ml as described previously,^21,42–45,48^. Briefly, viral samples were serially diluted 10-fold in complete medium and inoculated onto Huh7it-1 cells seeded on glass coverslips in a 24-well plate. After virus adsorption for 1 h, the cells were washed with medium to remove residual virus and cultured for 24 h. The virus-infected cells were washed with phosphate-buffered saline (PBS), fixed with 4% paraformaldehyde for 20 min and permeabilised with 0.1% Triton X-100 in PBS for 15 min at room temperature. After washing three times with PBS, the cells were incubated with a virus-specific primary antibody for 1 h, followed by incubation with FITC-conjugated respective secondary antibodies for 1 h. Then, the cells were observed under a fluorescence microscope. HIV-1 infectivity was determined by the TZM-bl assay using HIV-1 receptor-expressing HeLa cells, and the viral titres were expressed based on the β-galactosidase activity as reported previously^54^. MERS-CoV infectivity was determined by a FA method using a specific antibody^55^ on Vero cells 24 h after infection and also by a TCID_50_ assay on Vero cells by cultivating the virus-inoculated cells for 4 days, followed by fixing with phosphate-buffered formalin and staining with crystal violet^52^. The cells were cultivated in Dulbecco’s modified Eagle’s medium supplemented with 10% (for cell growth) or 2% (for maintenance culture) foetal bovine serum, non-essential amino acids, penicillin (100 IU/ml) and streptomycin (100 µg/ml) at 37 °C in a 5% CO_2_ incubator.

### Antibodies

The UV-inactivated anti-HCV human serum^42,43^ and rabbit antisera against FLUAV^21^, SeV^48^, HSV-1^49^ and MERS-CoV^55^ were described previously. A mouse monoclonal antibody against HCV NS3 (Millipore), rabbit polyclonal antibodies against DENV PrM (GeneTex), JEV NS3 (GeneTex) and glyceraldehyde-3-phosphate dehydrogenase (GAPDH; Millipore), rabbit antisera against CV-B3 (Denka Seiken) and horseradish peroxidase (HRP)-conjugated goat anti-mouse IgG and goat anti-rabbit IgG (Life Technologies) were purchased.

### Virucidal (neutralising) activity test

Cells were seeded into each well of a 6-well plate (plaque assay), 96-well plate (TCID_50_ test) or on a cover slip in each well of a 24-well plate (FA test). A fixed amount of test virus was mixed with serial dilutions of sPLA_2_s and incubated at 37 °C for 1 h. The mixtures were inoculated onto the cells and incubated for another 1 h to allow virus adsorption. Then, the cells were washed with medium to remove the residual virus and cultured for 2 to 4 days (plaque assay and TCID_50_ test), 24 h (FA test) or 2 days (TZM-bl assay) in fresh medium without sPLA_2_s. Viral solutions not treated with sPLA_2_s served as a control. The percent inhibition of viral infectivity by the sPLA_2_s compared to the untreated control was calculated, and the IC_50_ was determined.

### Time-of-addition test

CM-II-sPLA_2_ was added to the virus and/or cells at different time points relative to virus inoculation as described below. (i) Pretreatment of the cells with CM-II-sPLA_2_ before virus inoculation (to examine possible induction of an antiviral status in the cells). Huh7it-1 cells were treated with CM-II-sPLA_2_ for 1 h. After washing with medium to remove CM-II-sPLA_2_, the cells were inoculated with virus in the absence of CM-II-sPLA_2_ for another 1 h. After the residual virus was removed, the cells were cultured for 24 h in the absence of CM-II-sPLA_2_. (ii) Pretreatment of the virus with CM-II-sPLA_2_ followed by inoculation of the virus-CM-II-sPLA_2_ mixture onto the cells (to examine direct virucidal activity). The virus was incubated with CM-II-sPLA_2_ for 1 h, and the mixtures were inoculated onto Huh7it-1 cells. After 1 h of viral adsorption, the cells were washed with medium to remove the virus and CM-II-sPLA_2_, and cultured for 24 h in the absence of CM-II-sPLA_2_. (iii) Post-inoculation (post-entry) treatment of virus-infected cells with CM-II-sPLA_2_ (to examine post-entry antiviral activity in the infected cells). Huh7it-1 cells were inoculated with virus in the absence of CM-II-sPLA_2_. After 1 h of viral adsorption, the cells were washed with medium to remove the virus, and cultured for 24 h in the presence of CM-II-sPLA_2_. The viral titres in the inoculum and the degree of viral protein synthesis and viral RNA replication were determined by infectivity assay, immunoblotting and quantitative RT-PCR (qRT-PCR) analyses, respectively.

### Immunoblotting analysis

Immunoblotting was performed as reported previously^21^ with slight modifications. Briefly, Huh7it-1 cells were lysed in a Laemmli sample buffer (Bio-Rad) and the cell lysates were separated by 12% SDS-polyacrylamide gel electrophoresis and transferred to polyvinylidene difluoride membranes. The membranes were probed with the primary antibodies described above, followed by incubation with appropriate HRP-conjugated secondary antibodies. Bands were visualised using the Immobilon Western Chemiluminescent HRP Substrate (Millipore) and ImageQuant LAS 4000 (GE Healthcare).

### Quantitative RT-PCR (qRT-PCR)

The amounts of HCV RNA in the infected cells were determined by qRT-PCR, as described previously^53^. The amounts of DENV RNA were determined by TaqMan qRT-PCR, according to the protocol reported by Ito *et al*.^56^.

### Cytotoxicity assay

The cytotoxicity test was performed using the WST-1 reagent (Roche) as reported previously^21^. Briefly, Huh7it-1 cells plated in each well of a 96-well plate were treated with serial dilutions of sPLA_2_s at 37 °C for 24 h. Untreated cells served as a control. After this treatment, 10 µl of the WST-1 reagent was added to each well, and the cells were cultured for 4 h. The WST-1 reagent is converted to formazan by mitochondrial dehydrogenases; the amount of formazan was determined by measuring the absorbance of each well using a microplate reader at 450 and 630 nm. The percent cell viability compared to the untreated control was calculated for each dilution of sPLA_2_s, and the CC_50_ was determined. The LDH release assay was performed using a commercially available kit (Takara Bio) according to the manufacturer’s instructions.

### Haemolytic activity assay

The haemolytic activity was tested using human RBCs as reported previously^57,58^ with some modifications. Briefly, fresh human RBCs were washed three times with buffer (0.81% NaCl and 20 mM HEPES [pH 7.4]) and resuspended in the same buffer. A RBC suspension (10^7^–10^8^ RBCs/ml) was added to another buffer (0.81% NaCl, 20 mM HEPES [pH 7.4], and 2 mM CaCl_2_) containing various dilutions of sPLA_2_ (final volume = 100 μl). The mixtures were incubated at 37 °C for 1 h. After centrifugation, haemolysis was determined by measuring the absorbance of the supernatant at 570 nm. Controls for zero haemolysis and 100% haemolysis consisted of RBCs suspended in buffer and 0.5% Triton X-100 in buffer solution (or distilled water), respectively. The percentage of haemolysis was calculated for each dilution of sPLA_2_s, and the HC_50_ was determined.

## Electronic supplementary material

Supplementary Information
