# Supplementary material for: Broad-spectrum antiviral agents: secreted phospholipase A2 targets viral envelope lipid bilayers derived from the endoplasmic reticulum membrane
Source: Sci Rep. 2017 Nov 21;7:15931. doi: 10.1038/s41598-017-16130-w (PMC5698466; doi:10.1038/s41598-017-16130-w)
Supplement: Supplementary file 1 — Supplementary Information [file 41598_2017_16130_MOESM1_ESM.pdf]

Broad-spectrum antiviral agents: secreted phospholipase A_2 targets viral envelope lipid bilayers derived from the endoplasmic reticulum membrane

Ming Chen[1], Chie Aoki-Utsubo[2], Masanori Kameoka[2], Lin Deng[3], Yutaka Terada[4], Wataru Kamitani[4], Kei Sato[5,6], Yoshio Koyanagi[5], Makoto Hijikata[7], Keiko Shindo[8], Takeshi Noda[8], Michinori Kohara[9] & Hak Hotta[1,*]

[1]Department of Vaccine and Drug Development, Graduate School of Health Sciences, Kobe University, Kobe 650-0047, Japan

[2]Department of International Heath, Graduate School of Health Sciences, Kobe University, Kobe 654-0147, Japan

[3]Division of Infectious Disease Control, Graduate School of Medicine, Kobe University, Kobe 650-0017, Japan

[4]Laboratory of Clinical Research on Infectious Diseases, Research Institute for Microbial Diseases, Osaka University, Suita, Osaka 565-0871, Japan

[5]Laboratory of Systems Virology, Institute for Frontier Life and Medical Sciences, Kyoto University, Kyoto 606-8507, Japan

[6]CREST, Japan Science and Technology Agency, Saitama 322-0012, Japan

[7]Laboratory of Tumour Viruses, Institute for Frontier Life and Medical Sciences, Kyoto University, Kyoto 606-8507, Japan

[8]Laboratory of Ultrastructural Virology, Institute for Frontier Life and Medical Sciences, Kyoto University, Kyoto 606-8507, Japan

[9]Infectious Disease Regulation Project, Tokyo Metropolitan Institute of Medical Science, Tokyo 156-8506, Japan

*Correspondence and request for materials should be addressed to H.H. (email: hotta@kobe-u.ac.jp)

Supplementary Table S1. Time-of-addition experiments using CM-II-sPLA$_2$ against HCV and DENV.

Virus	CM-II-sPLA$_2$ (ng/ml)	% Reduction of the number of virus-infected cells		
		Pretreatment of cells	Pretreatment of virus	Post-entry treatment
HCV	1,000	93 ± 1	>99	<1
	100	54 ± 3	>99	<1
	10	16 ± 1	>99	<1
	1	4 ± 1	>99	<1
DENV	1,000	96 ± 1	>99	<1
	100	83 ± 5	>99	<1
	10	37 ± 3	>99	<1
	1	2 ± 2	>99	<1

Data are presented as the average ± SEM (n = 2).

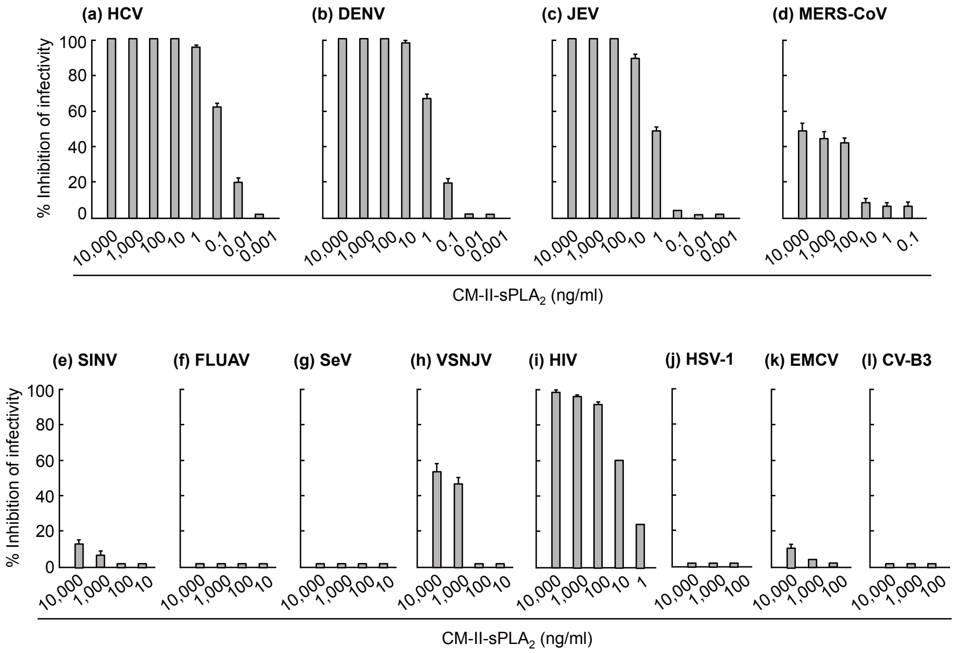

Supplementary Figure S1. Dose-dependent inhibition of a panel of viruses by CM-II-sPLA$_2$. A fixed amount of test virus was mixed with serial dilutions of sPLA$_2$s and incubated at 37°C for 1 h. The mixtures were inoculated onto the cells and incubated for another 1 h to allow virus adsorption. Then, the cells were washed with medium to remove the residual virus and cultured for 2 to 4 days (plaque assay and TCID$_{50}$ test), 24 h (FA test) or 2 days (TZM-bl assay) in fresh medium without sPLA$_2$s. Viral solutions not treated with sPLA$_2$s served as a control. The percent inhibition of viral infectivity by the sPLA$_2$s compared to the untreated control was calculated. Data are presented as the average ± SEM (n = 3 to 5).

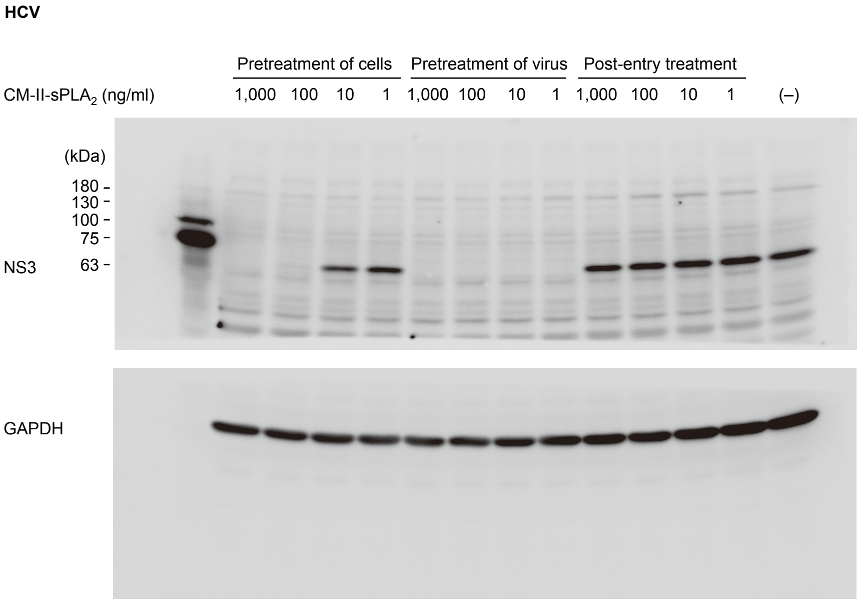

Supplementary Figure S2. Time-of-addition experiments using CM-II-sPLA$_2$ against HCV. HCV NS3 expression levels were examined by immunoblotting analysis. (i) Pretreatment of the cells: Huh7it-1 cells were treated with various concentrations of CM-II-sPLA$_2$ (1,000, 100, 10 and 1 ng/ml) for 1 h. Then, the cells were inoculated with HCV in the absence of CM-II-sPLA$_2$ for another 1 h and cultured for 24 h in the absence of CM-II-sPLA$_2$. (ii) Pretreatment of the virus: HCV was incubated with CM-II-sPLA$_2$ for 1 h, and the mixtures were inoculated to Huh7it-1 cells. After 1 h, the cells were cultured for 24 h in the absence of CM-II-sPLA$_2$. (iii) Post-entry treatment: Huh7it-1 cells were inoculated with HCV in the absence of CM-II-sPLA$_2$. After 1 h, the cells were cultured for 24 h in the presence of CM-II-sPLA$_2$. (–), Untreated control. The lysates of the infected cells were subjected to 12% SDS-polyacrylamide gel electrophoresis and the full-length gel was electrophoretically blotted onto a polyvinylidene difluoride membrane. The membrane was cut into two pieces, which were probed with either anti-HCV NS3 (upper panel) or anti-GAPDH antibodies (lower panel). GAPDH bands serve as a loading control.

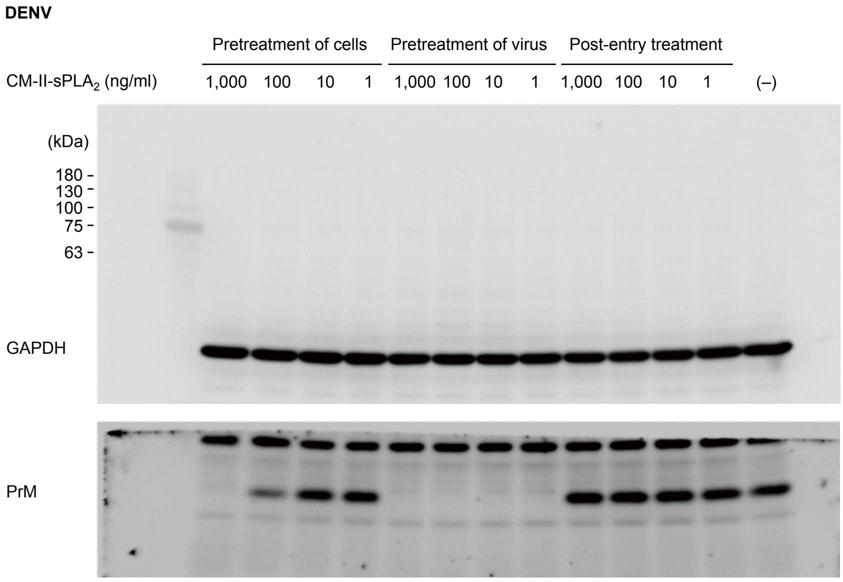

Supplementary Figure S3. Time-of-addition experiments using CM-II-sPLA$_2$ against DENV. DENV PrM expression levels were examined by immunoblotting. (i) Pretreatment of the cells: Huh7it-1 cells were treated with various concentrations of CM-II-sPLA$_2$ (1,000, 100, 10 and 1 ng/ml) for 1 h. Then, the cells were inoculated with DENV in the absence of CM-II-sPLA$_2$ for another 1 h and cultured for 24 h in the absence of CM-II-sPLA$_2$. (ii) Pretreatment of the virus: DENV was incubated with CM-II-sPLA$_2$ for 1 h, and the mixtures were inoculated onto Huh7it-1 cells. After 1 h, the cells were cultured for 24 h in the absence of CM-II-sPLA$_2$. (iii) Post-entry treatment: Huh7it-1 cells were inoculated with DENV in the absence of CM-II-sPLA$_2$. After 1 h, the cells were cultured for 24 h in the presence of CM-II-sPLA$_2$. (–), Untreated control. The lysates of the infected cells were subjected to 12% SDS-polyacrylamide gel electrophoresis and the full-length gel was electrophoretically blotted onto a polyvinylidene difluoride membrane. The membrane was cut into two pieces, which were probed with either anti-GAPDH (upper panel) or anti-DENV PrM antibodies (lower panel). GAPDH bands serve as a loading control.
